# Preventing Cardiometabolic Disease in HIV-Infected Latino Men: The Happy Older Latinos Health Promotion Study

**DOI:** 10.1093/geroni/igaa057.2290

**Published:** 2020-12-16

**Authors:** Daniel Jimenez

**Affiliations:** University of Miami, Coral Gables, Florida, United States

## Abstract

Older Latinos living with HIV have been disproportionately affected by the epidemic and experience compounded health disparities that have deepened over time. Eighteen Latinos living with HIV with a mean age of 60.3 years (SD=6.4) were enrolled in the Happy Older Latinos are Active (HOLA), a community health worker-led, multicomponent, health promotion intervention. Participants were assessed at three time points on measures of cardiometabolic risk and psychosocial functioning. We evaluated the feasibility of recruitment, retention, acceptability, and implementation of HOLA. In 4 months, we met our enrollment target with <5% of eligible participants refusing participation. Participants attended over 70% of sessions and 1 participant was lost to follow up. These results indicate that HOLA is an innovative health promotion program that is uniquely tailored to address the multiple concerns that are prevalent in this community (cardiometabolic risk, psychological distress) in a nonstigmatizing and culturally acceptable manner.

